# Clinical management of autoimmune hepatitis

**DOI:** 10.1177/2050640619872408

**Published:** 2019-08-25

**Authors:** Simon Pape, Christoph Schramm, Tom JG Gevers

**Affiliations:** 1 Department of Gastroenterology and Hepatology, Radboud University Medical Center, Nijmegen, The Netherlands; 2 European Reference Network Hepatological Diseases (ERN RARE-LIVER), Hamburg, Germany; 3 1st Department of Internal Medicine, University Medical Center Hamburg-Eppendorf, Hamburg, Germany; 4 Martin Zeitz Centre for Rare Diseases, University Medical Centre Hamburg-Eppendorf, Hamburg, Germany

**Keywords:** Autoimmune hepatitis, prednisone, prednisolone, induction therapy, European Association for Study of the Liver, clinical management

## Abstract

Autoimmune hepatitis is a rare and chronic liver disease that is characterised by increased serum transaminases and immunoglobulin G, inflammatory liver histology and presence of circulating autoantibodies. An autoimmune hepatitis diagnosis justifies life-long treatment in most patients in order to prevent development of cirrhosis and end-stage liver disease. The cornerstone of treatment is steroid induction therapy followed by maintenance therapy with azathioprine, which is effective in most cases. For patients who do not respond to standard treatment, second-line treatment with other immunosuppressants can be effective. Treatment should be aimed at biochemical remission of the disease, which is defined as normalization of transaminases and immunoglobulin G. Patients should be monitored intensively during the first months of treatment in order to monitor side-effects, assess symptoms and individualise treatment. Specialist consultation should be sought in difficult-to-treat patients. Future studies and networking initiatives should result in optimization of current treatment strategies in autoimmune hepatitis.

## Introduction

Autoimmune hepatitis (AIH) is a chronic inflammatory liver disease that predominantly affects women, but can occur in all ages and races. The exact cause of AIH is unknown, although it is hypothesised that loss of tolerance against liver antigens is the main pathophysiological mechanism, which is triggered by environmental factors in individuals with a certain genetic susceptibility.^1^ AIH is characterised by hypergammaglobulinaemia, circulating auto-antibodies and distinctive histology. Based on these characteristics, the International Autoimmune Hepatitis Group (IAIHG) has established diagnostic criteria (Table 1) that aid physicians in establishing a correct AIH diagnosis. However, a diagnosis of AIH remains a clinical one, since a gold standard for diagnosis is lacking.^2,3^

**Table 1.** Simplified diagnostic criteria for the diagnosis of autoimmune hepatitis (AIH).

Variable	Cut-off	Points
ANA or SMA	Titre ≥ 1:40	1
ANA or SMA	Titre ≥ 1:80	2
Or LKM1	≥1:40	2
Or SLA/LP	Any titre	2^a^
IgG	>ULN	1
	>1.1 × ULN	2
Liver histology (evidence of hepatitis is a necessary condition)	Atypical	0
	Compatible with AIH	1
	Typical for AIH	2
Absence of viral hepatitis	Yes	2
	No	0
	Probable AIH	≥6
	Definite AIH	≥7

ANA: anti-nuclear antibody; IgG: immunoglobulin G; LKM1: liver kidney microsomal type 1 antibody; SLA/LP: anti-soluble liver antigen/liver-pancreas antibody; ULN: upper limit of normal.

a Addition of points achieved for all autoantibodies (two points maximum). Typical liver histology for AIH includes each of the following features: interface hepatitis, lymphocytic infiltrates in the portal tracts and extended into the lobule, emperipolesis (active penetration by one cell into and through a larger cell) and hepatic rosette formation. Compatible liver histology includes: chronic hepatitis with lymphocytic infiltration without the features considered typical. Atypical histology includes signs of other liver diseases such as steatohepatitis.

Even though establishing the diagnosis of AIH is sometimes complex and cumbersome, clinical management of AIH can also be a challenging journey, given the lifelong therapy and potential side-effects. In this review article, we will discuss the clinical management of adult AIH patients and its latest developments, based on recent literature. Our aim is to assist the general gastroenterologist and hepatologist in the management of AIH, once an AIH diagnosis has been confirmed.

## Literature search

We performed a PubMed search with the MeSH term ‘autoimmune hepatitis’ and ‘autoimmune hepatitis’ in the title field. All searches were limited to the English language and publication date within the last five years at time of search (May 2019). For the purpose of this review, we primarily selected articles that focus on clinical management of AIH. For a comprehensive review on mechanisms and diagnosis of AIH we refer to another article.^1^ We identified a total of 114 articles that met our inclusion criteria.

## Why should we treat an AIH patient?

Typically, the first question of AIH patients after hearing their diagnosis is: ‘Do I need treatment?’. Untreated AIH leads to progression of fibrosis to cirrhosis and, eventually, end-stage liver disease. Older studies showed that immunosuppressive treatment with steroids in AIH patients not only improved liver function tests, but also improved symptoms and prolonged survival.^4–6^ More recent studies have shown that treatment also leads to regression of liver fibrosis, even at the cirrhotic stage of disease.^7^ This data indicates that treatment is warranted in patients with AIH.

## Treatment for everyone?

It is unknown whether patients with mild disease (ALT < 3 times upper limit of normal, histological activity index (HAI) < 3 and no advanced fibrosis) will benefit from treatment, since most studies only included patients with moderate to severe disease activity. A decision not to treat mild AIH can be deemed as a possible option, especially in patients of older age or with severe comorbidities. However, AIH has a fluctuating disease course and patients who present asymptomatically may develop symptoms or elevation of transaminases that warrant treatment.^8^ Therefore, we recommend treatment in every AIH patient, unless there are compelling reasons not to treat. Without treatment, close monitoring of transaminases and immunoglobulin G (IgG) should occur every 3–6 months in order to detect a possible flare of the disease and non-invasive measures of liver fibrosis such as transient elastography (TE) can be used to monitor for disease progression.

## Is AIH treatment lifelong?

Immunosuppressive therapy should be continued for at least two years following complete normalization of transaminases and IgG. One study found that relapse of the disease occurs in up to 90% of patients once treatment is stopped.^9^ In patients eligible for a trial of drug withdrawal, liver enzymes should be monitored closely. Using this approach, one study reported a long-term remission rate of 54% after drug withdrawal.^10^ Preferably, a liver biopsy should be performed prior to stopping of therapy. If there is histological disease activity (HAI > 3) present, immunosuppressive treatment should not be stopped. The HAI is a histological scoring tool that rates the hepatitis components of periportal necrosis, intralobular degeneration and portal inflammation on a scale from 1–18. A HAI score from 1–3 indicates minimal hepatitis (Supplementary Material Table 1).^11^

## How should we treat an AIH patient?

### Steroid induction therapy

Steroid therapy is the mainstay for inducing remission in AIH: studies with azathioprine induction therapy alone showed low remission rates and high mortality.^12^ Steroid treatment, predniso(lo)ne in most cases, can be initiated as monotherapy or in combination with azathioprine.^13^ Most guidelines advise an initial predniso(lo)ne dose between 0.50–1.00 mg/kg per day, although some centres start with a high initial dose of 1.00 mg/kg with rapid tapering within the following months.^14,15^ A recent retrospective study showed that patients who were treated with a predniso(lo)ne dose below 0.50 mg/kg/day, achieved similar remission rates when compared to patients who were treated with higher dosages.^16^ Given the considerable side-effects of steroid therapy, physicians should prescribe a predniso(lo)ne dose that provides ample suppression of inflammatory activity and that is acceptable to the individual patient in terms of tolerability. We prefer tapering of steroids to be response-guided and tailored to the individual patient, in contrast to a fixed steroid-dosing schedule, although both methods have never been compared in AIH.

Budesonide at a dose of 9 mg/day provides an alternative induction agent in AIH and is associated with less steroid-related side-effects, but is contraindicated in patients with cirrhosis due to increased systemic side-effects as a result of portosystemic shunting.^17^

### Maintenance therapy

**Figure 1.**
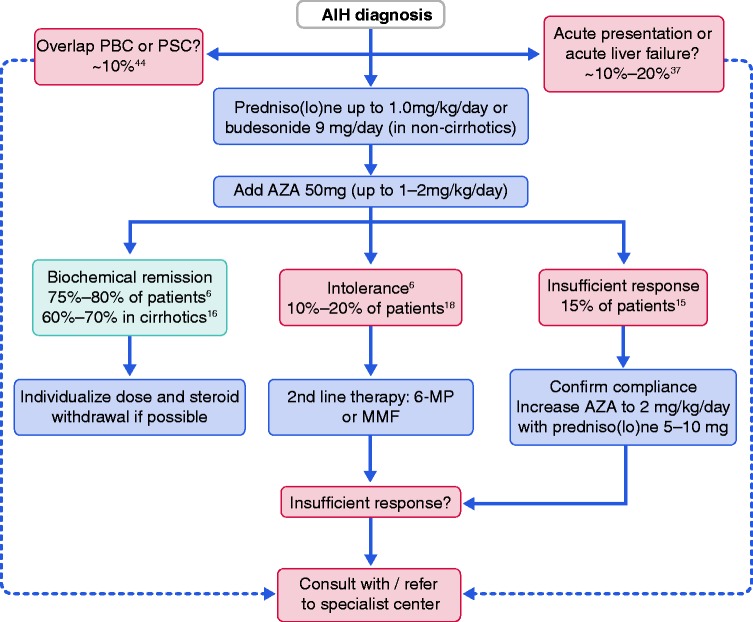
Treatment algorithm for an adult patient with a first presentation of autoimmune hepatitis (AIH). The mainstay of treatment is steroid induction therapy followed by maintenance therapy with azathioprine (AZA). AIH treatment should always be individualised. 6-MP: 6-mercaptopurine; MMF: mycophenolate mofetil; PBC: primary biliary cholangitis; PSC: primary sclerosing cholangitis.

Azathioprine is the first drug of choice for maintenance therapy in AIH.^18^ Azathioprine is ideally introduced 2–4 weeks after initiation of steroid treatment, in order to anticipate possible hepatotoxicity. To minimise side-effects, azathioprine is started at a dose of 50 mg/day, which can be increased to 1–2 mg/kg/day, depending on individual treatment response (Table 2). Both a combination of predniso(lo)ne and azathioprine and azathioprine alone are effective in maintaining remission,^6^ although tapering steroids should occur as soon as maintenance therapy is initiated. With this regimen, 75–80% of patients will achieve normalization of transaminases.^19^ Steroid-free therapy should be a treatment goal in every AIH patient to prevent steroid-related complications and should be aimed for within the first year of treatment. There is not sufficient data to recommend an alternative to azathioprine as first-line therapy. Mycophenolate mofetil (MMF) has proven to be effective and safe as first-line treatment in uncontrolled studies, with remission rates up to 88% and dosages from 1000–2000 mg/day.^20,21^ A simple algorithm for AIH treatment is presented in Figure 1.

**Table 2.** Key recommendations for treatment of an adult autoimmune hepatitis (AIH) patient.

Treatment is indicated in every patient and is generally life-long.
Steroid induction therapy with predniso(lo)ne or budesonide is needed to induce remission.
Azathioprine is the first drug of choice for maintenance of remission.
Tapering of steroids should be response guided and tailored to the individual patient.
Treatment should be aimed at biochemical remission: normalization of ALT/AST and IgG.
Patients with side-effects on azathioprine might benefit from a switch to 6-MP.
Treatment with MMF or CNIs should only be done by physicians with experience.
Patients with cirrhosis should undergo hepatocellular carcinoma surveillance.

6-MP: 6-mercaptopurine; IgG: immunoglobulin G; MMF: mycophenolate mofetil.

## What is a satisfactory response during treatment?

### Remission of disease

Histological and biochemical remission of AIH should be the fundamental treatment goal in every patient. A HAI score of <4/18 is used to define histological remission. Since frequent biopsies are an unattractive option, the surrogate endpoint of biochemical remission is used, which is defined as repeatedly normal serum transaminases and normal serum IgG. This endpoint is incorporated in most international guidelines.^13,22^ A recent study demonstrated that biochemical remission is associated with regression of fibrosis and low histological disease activity.^7^ A different study showed that histological hepatitis activity still exists in approximately 50% of patients even though they had achieved biochemical remission.^23^ Additionally, AIH is a disease with an unpredictable course and frequent relapses. Therefore, AIH patients should be monitored for transaminases and IgG at six-month intervals at least even if they are in prolonged biochemical remission. Use of TE may be helpful for the detection of fibrosis progression during the disease course.^24^

## What problems do we encounter during AIH treatment?

### Side-effects

Steroid therapy is accompanied by a variety of side-effects, including weight gain, diabetes mellitus, hypertension, emotional instability and even psychosis, that necessitate dose reduction or withdrawal of the drug. Steroid-related side effects occur in 80% of patients after two years of treatment,^5,25^ although this occurs less often in combination therapy with azathioprine.^6^ Measurement of bone density is recommended at the initiation of steroid therapy and patients should receive vitamin D supplementation and adequate dietary calcium in order to prevent the development of osteoporosis (Table 3).

**Table 3.** Management of medication and side-effects.

Medication	Side-effect	How to manage?
Steroids	Diabetes mellitus	Regular glucose measurements at start of steroid therapy HbA1c monitoring 6–12 monthly
	Osteoporosis	Bone densitometry at start of steroid therapy and at 1–5 year intervals Supplementation of vitamin D and adequate calcium intake Bisphosphonates in patients with osteoporosis
	Cataract	Ophthalmic assessment when on long-term steroids
	Hypertension	Blood pressure assessment in patients with documented hypertension
Azathioprine	Cytopaenia	Full blood count measurements every 2–4 weeks after start of treatment, followed by three-month intervals
	Non-melanoma skin cancer	UV protective measures Dermatological monitoring when on long-term treatment

The exact number of AIH patients that experience azathioprine intolerance is unknown in AIH, but old series report rates of 10–20%, although this may be higher in real-world practice.^13,25^ Most side-effects are limited to nausea and gastrointestinal discomfort but in some cases may result in rash, arthralgia or pancreatitis which warrants discontinuation.^15^ Split-dose administration of azathioprine is an easy option to diminish mild side-effects.^26^ Full blood counts should be monitored to detect possible bone marrow toxicity. Some patients might benefit from dose reduction, although close monitoring of transaminases and IgG is recommended to avert a possible flare of the disease. In case of intolerable side-effects, alternative immunosuppression in recommended.

### Insufficient response to treatment

Approximately 15% of patients may experience an insufficient response to therapy by demonstrating failure to normalise biochemical or histological parameters. A first step should always be to reconfirm the AIH diagnosis, preferably with help of an experienced liver pathologist. Second, it is important to ensure treatment adherence, given that adherence rates can drop to 65% after six months of chronic drug therapy in other chronic diseases.^27^ Measuring 6-thioguanine (6-TG) concentrations during treatment may help to identify those patients who are non-adherent. Little is known about optimal 6-TG concentrations, although one study showed that 6-TG concentrations above 220 pmol/8 × 10^8^ RBC are associated with remission.^28^ Patients with an insufficient response to standard therapy might benefit from increased dosages of azathioprine up to 2 mg/kg/day, together with 5–10 mg predniso(lo)ne.

### Alternative treatment options

There is no consensus on the best second-line treatment options in AIH. Intolerance and insufficient response are two different scenarios. In general, intolerance to treatment is manageable with 6-mercaptopurine (6-MP) or MMF, while an insufficient response to first-line treatment is more difficult to deal with.

6-MP is widely used as an alternative to azathioprine in inflammatory bowel disease (IBD) and has better tolerability.^29^ Although it is used in clinical management of AIH, data on 6-MP efficacy is limited to two case series with a dosage ranging from 25–75 mg/day.^30,31^ It shows a favourable response especially in azathioprine-intolerant patients, and acceptable tolerance rates. 6-TG can also be used as an alternative to either azathioprine or 6-MP but has been associated with development of nodular regenerative hyperplasia in patients with IBD.^32^ A recent study in AIH patients showed that 6-TG treatment with a dose of 20 mg/day is well-tolerated and leads to complete biochemical remission in patients with prior insufficient response to thiopurines.^33^

The most studied second-line drug in AIH is MMF at a dose of 1000–2000 mg/day. Reduction of serum transaminases occurs in 33–100% of patients and histological remission occurs in 73% of patients.^34^ A recent study showed that patients on MMF with an initial non-response to first-line treatment have lower remission rates (34–57%) than patients with azathioprine intolerance (62–91%).^35^ MMF has a favourable safety profile but has teratogenic effects, which makes the drug less useful in women in childbearing age. We recommend that treatment with MMF should be provided by physicians who have experience with the drug.

The calcineurin inhibitors tacrolimus and cyclosporine are options for second-line therapy in AIH. Reports on these drugs demonstrate response rates ranging from 27–94%.^36^ Calcineurin inhibitors can have considerable side effects with nephrotoxicity and hypertension being the most prominent. Prescription should therefore only be carried out by physicians with ample experience concerning these drugs.

## How to manage difficult-to-treat AIH patients?

### Acute presentation

There are no validated definitions for AIH with acute presentation.^37^ Patients who present with acute, icteric AIH and concomitant coagulopathy (INR≥1.5) respond to oral or intravenous steroids (1 mg/kg) in the majority of cases, although in a number of patients transplantation might be the only treatment option.^38–40^

However, steroid use might not be beneficial in the setting of acute liver failure with hepatic encephalopathy, one retrospective study found that corticosteroid use in AIH with acute liver failure was associated with increased mortality in patients with the highest MELD score.^41^ Timely consultation with a transplant centre is strongly recommended to arrange rapid referral when necessary.

### Patients with cirrhosis

Up to 30% of patients have confirmed cirrhosis at diagnosis, which is a sign of subclinical disease course months or even years prior to diagnosis and is associated with poorer outcomes.^8,19,42^ Patients who present with decompensated cirrhosis should be treated in close collaboration with a transplant centre. It is advised to offer ultrasound surveillance to cirrhotic patients.^43^

### Variant syndromes with primary biliary cholangitis (PBC) and primary sclerosing cholangitis (PSC)

AIH patients might present with additional features of PBC or PSC, or develop these features at a certain time during their disease course. Standardised definitions of these variant syndromes are currently lacking and, given their rarity, evidence-based recommendations for treatment are lacking as well. In general, it is important to treat patients with autoimmune liver disease according to their predominant phenotype.^44^ Consultation with an expert centre is advised in order to prevent overtreatment.

### Mood disorders

Improvement in mood disorders should be an important treatment goal in AIH. Depressive symptoms and anxiety are often present in AIH patients and results in lower health-related quality of life (HRQoL), even when in biochemical remission.^45^ Since occurrence of depressive and anxiety symptoms in AIH may be associated with non-adherence to treatment,^46^ physicians should actively ask patients about possible symptoms of depression and anxiety and refer them to mental healthcare providers if necessary. Although improvement of HRQoL should be a desirable treatment goal in AIH, validated questionnaires that assess the wide range of problems in AIH are currently lacking.

## When should we refer an AIH patient to a specialist centre?

Timely consultation with an expert centre and/or transplant centre is recommended in case of diagnostic uncertainties, patients with features of variant syndromes, pregnant patients and issues regarding optimal disease management.^15^ For patients who fail to demonstrate a sufficient biochemical response after use of a second immunosuppressant or when the treating physician is unfamiliar with prescribing second-line therapy, expert advice should be sought. Finally, patients who present with acute liver failure and are eligible for liver transplantation should be transferred to a transplant centre (Table 4).

**Table 4.** Scenarios in which consultation with a specialist centre is recommended.

Uncertainties regarding diagnosis: seek help from an expert liver pathologist
Uncertainties regarding treatment indication (e.g. old age/low disease activity)
Presentation with acute fulminant hepatitis^a^
Signs of acute liver failure (severe coagulopathy, hepatic encephalopathy)^a^
Insufficient response after a second immunosuppressant or planned treatment with MMF or calcineurin inhibitors and insufficient experience with the drugs
Patients with additional features of PBC or PSC
Pregnancy
Planned cessation of therapy

MMF: mycophenolate mofetil; PBC: primary biliary cholangitis; PSC: primary sclerosing cholangitis.

a Consultation with transplant centre is strongly recommended.

The European Reference Network for Hepatological Diseases (ERN RARE-LIVER) was launched in March 2017 and is a European Union credited joint venture between expert centres across Europe, linking professional societies and patients' groups with the main objective to improve the care of patients with rare liver diseases.^47^ Aside from conducting prospective high-quality registries, ERN RARE-LIVER offers expert advice on difficult AIH cases using an online clinical patient management system established by EU. These developments will undoubtedly lead to optimization of current treatment strategies and to reduce discrepancies in AIH care delivery.

## Future prospects

### Drugs under investigation

Currently, a trial is ongoing with ianalumab, a monoclonal antibody against the B-cell activating factor receptor^48^ (Table 5). Other experimental strategies are aimed at increasing the pool and function of regulatory T-cells,^49^ for example by subcutaneous low-dose interleukin-2, which has proved to reduce inflammatory liver damage.^50,51^ A phase I trial with subcutaneous synthetic preimplantation factor showed good safety and tolerability in AIH patients, but was not continued into phase II (source: NCT03593460).^52^

**Table 5.** Ongoing trials with new drugs in autoimmune hepatitis (AIH).

Study name/NCT number	Study drug	Treatment target	No. of patients	Primary endpoint
AMBER/NCT03217422	VAY736/ianalumab	B-cell activating factor	80	ALT normalization after 24 weeks
NCT02556372	JKB-122	Toll-like receptor 4	20	Changes in ALT levels after 24 weeks
MERLIN/NCT02997878	Mesenchymal stromal cells	Various immunomodulatory properties	56	Dose finding/safety

## Conclusion

AIH treatment involves steroid induction therapy followed by maintenance therapy with azathioprine with biochemical remission of disease as primary treatment goal, which is achievable for the majority of patients. Caution should be exercised in patients with an acute, fulminant presentation of the disease, in cirrhotics, pregnancy and in patients with prolonged insufficient response to standard therapy. Early access to specialist consultation should improve AIH healthcare delivery and outcome. Referral should take place for difficult cases. International networks such as the ERN RARE-LIVER will provide groundwork for registries and studies that will provide more insight into optimal management strategies in AIH.

## Supplemental Material

Supplemental material for Clinical management of autoimmune hepatitis Click here for additional data file. Supplemental Material for Clinical management of autoimmune hepatitis by Simon Pape, Christoph Schramm and Tom JG Gevers in United European Gastroenterology Journal
