# Supplementary material for: Clinical management of autoimmune hepatitis
Source: United European Gastroenterol J. 2019 Aug 25;7(9):1156–63. doi: 10.1177/2050640619872408 (PMC6826525; doi:10.1177/2050640619872408)
Supplement: Supplemental material for Clinical management of autoimmune hepatitis [file Supplemental3_Material.pdf]

Supplementary table 1

**Periportal ± bridging necrosis**	**Score**	**Intralobular degeneration and focal necrosis**	**Score**	**Portal inflammation**	**Score**	**Fibrosis**	**Score**
None	0	None	0	No portal inflammation	0	No fibrosis	0
Mild piecemeal necrosis	1	Mild (acidophilic bodies, ballooning, degeneration and/or scattered foci of hepatocellular necrosis in <1/3 of lobules or nodules	1	Mild (sprinkling of inflammatory cells in <1/3 of portal tracts)	1	Fibrous portal expansion	1
Moderate piecemeal necrosis (involves less than 50 percent of the circumference of most portal tracts)	3	Moderate (involvement of 1/3 to 2/3 of lobules or nodules)	3	Moderate (increased inflammatory cells in 1/3 to 2/3 of portal tracts)	3	Bridging fibrosis (portal-portal or portal-central linkage)	3
Marked piecemeal necrosis (involves more than 50 percent of the circumference of most portal tracts)	4	Marked (involvement of >2/3 lobules or nodules)	4	Marked (dense packing of inflammatory cells in >2/3 of portal tracts)	4	Cirrhosis	4
Moderate piecemeal necrosis plus bridging necrosis	5						
Marked piecemeal necrosis plus bridging necrosis	6						
Multilobular necrosis	10						

Histological activity index (HAI) for chronic hepatitis. The composite score is based on histologic assessment of periportal and/or bridging necrosis, intralobular degeneration and focal necrosis, portal inflammation, and fibrosis. A HAI (without the fibrosis component) of 1 – 3 indicates minimal hepatitis and is seen as histological remission in AIH. A score of 4 – 8 corresponds with mild hepatitis, 9 – 12 with moderate hepatitis, and 13 – 18 with severe hepatitis.
